# Clinical Insights and Advancements in Human Metapneumovirus Management and Prognosis

**DOI:** 10.15190/d.2025.3

**Published:** 2025-03-31

**Authors:** Harsahaj Singh Wilkhoo, Afra Wasama Islam, Harkirat Singh Wilkhoo, Suhaib Hussain, Bharat Singh, Saumya Rajesh Kadam

**Affiliations:** ^1^Faculty of Medicine, Tbilisi State Medical University, Tbilisi, Georgia; ^2^ClinNova International, Tbilisi, Georgia; ^3^Arabian Healthcare Group, Ras Al Khaimah, United Arab Emirates

**Keywords:** Human metapneumovirus, public health, Ribavirin, Monoclonal antibodies, Immunomodulator.

## Abstract

Human metapneumovirus is a respiratory pathogen that infects children, the elderly, and immunocompromised individuals. Despite its global prevalence, underdiagnosis persists because of clinical overlap with other respiratory viruses. The current approach is mostly supportive, with oxygen therapy and hydration being crucial interventions. Ribavirin contains antiviral properties but has little clinical application. Vaccine development is moving forward, with prospects including live-attenuated, subunit-based, and virus-like particle vaccines. Molecular diagnostics, such as RT-PCR and metagenomic sequencing, have increased detection rates, which aids epidemiological monitoring. Monoclonal antibodies targeting the fusion (F) protein are being studied for passive immunity, while immunomodulatory treatments such as corticosteroids and intravenous immunoglobulins may help treat severe cases. Emerging treatments include fusion inhibitors and pan-pneumovirus vaccinations that protect against HMPV and RSV. Future research should concentrate on optimizing antiviral methods, increasing vaccination trials, and improving surveillance to detect outbreaks. A multidisciplinary approach that combines virology, immunology, and epidemiology is required to reduce HMPV's effect and improve patient outcomes. This review serves as a comprehensive literature about HMPV which provides all the crucial clinical perspectives and the latest advancements in management, antivirals, patient prognosis as well and diagnostic modalities.

## SUMMARY

1. Introduction

2. Epidemiology and Disease Burden

3. Virology and Pathogenesis

4. Clinical Manifestations and Complications

5. Diagnostic approaches

6. Current management options

7. Vaccines and Preventive Measures

8. Future Directions in outbreak prevention and management

9. Conclusion

## 1. Introduction

Human Metapneumovirus (HMPV), a paramyxo-virus, is an emerging respiratory pathogen that has received significant attention due to its impact on patient outcomes and therapeutic advances. HMPV is a non-segmented, single-stranded RNA virus from the Pneumoviridae family. It was first discovered in the Netherlands in 2001 and is now recognized as a major cause of respiratory tract infections in individuals of all ages, particularly children, the elderly, and those who are immunocompromised. It is a seasonal virus that normally peaks in the winter and early spring, much like respiratory syncytial virus (RSV) and influenza. It is made up of two genetic groups, A and B, with subtypes A1, A2, B1, and B2, which change annually. Clinically, HMPV can cause a wide range of respiratory disorders, from minor upper respiratory tract infections like cough and nasal congestion to severe lower respiratory conditions like bronchiolitis and pneumonia ^1,2^. Advances in diagnostic technologies, notably Polymerase Chain Reaction-based assays, have greatly improved HMPV identification and comprehension. There is presently no specific antiviral therapy or vaccination available. The primary treatment is supportive, which includes hydration and oxygen therapy, while experimental antiviral treatments are being studied in clinical trials^2,3^.

In December 2024, an increase in HMPV cases was seen, mainly in China, where it was associated with 6.2% of positive respiratory tests and 5.4% of hospitalizations. The World Health Organization attributes the increase to seasonal fluctuations rather than a new pandemic threat. Given that HMPV disproportionately affects vulnerable individuals, ongoing surveillance is critical for early detection and outbreak control. The ongoing study is to create targeted medicines and vaccinations to lessen the global burden of HMPV infections ^4-6^.

## **2.** Epidemiology and Disease Burden

HMPV is found all across the world, and its peak activity corresponds with that of RSV. The virus is prevalent in late winter and spring, from January to March in the northern hemisphere and from June to July in the southern hemisphere, despite its year-round circulation. Other criteria, such as the child's age and the prevalence of other chronic illnesses, ultimately determine the severity of the disease, which can range from moderate respiratory symptoms to severe pneumonia ^4,7^.

Compared to other similar respiratory viruses like RSV, it was found that children who were born prematurely or who had any other chronic airway illness, such as asthma, were more likely to contract HMPV. The severity of the sickness was similarly associated with the child's age. In comparison to other age groups, children between the ages of 12 and 23 months displayed the most severe symptoms, leading to the conclusion that a child under the age of 6 months is significantly protected by maternal antibodies ^8^. Although re-infection can happen throughout adulthood, multiple seroprevalence studies have revealed that a significant portion of children (90–100%) had been infected by the time they were 5–10 years old. The highest age of hospitalization for HMPV is between 6 and 12 months, according to numerous studies; this is later than the peak age of hospitalization for RSV, which is between 2 and 3 months ^5,9^.

**Figure 1:**
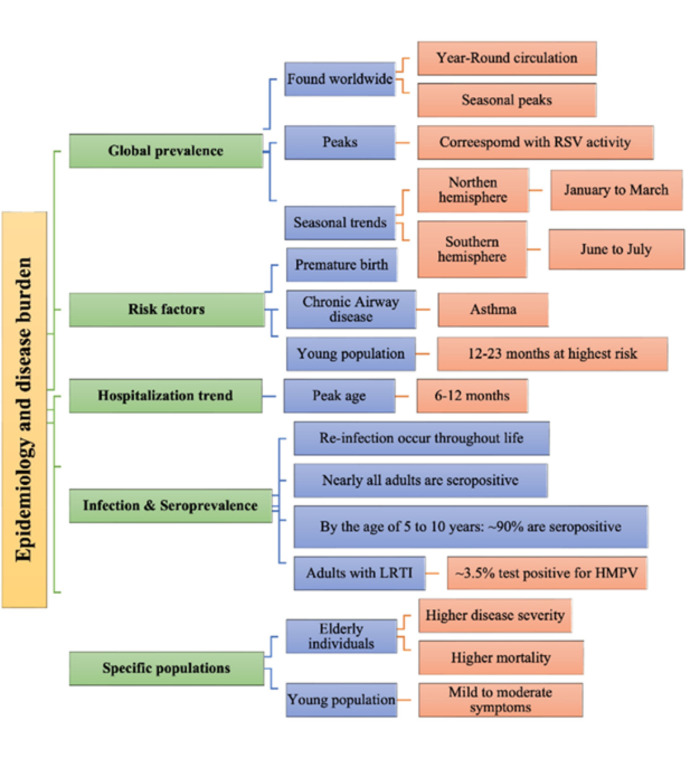
Figure 1. Epidemiology and disease burden of human metapneumovirus

Although it occurs at a younger rate - roughly 3.5% of adults with LRTI test positive for HMPV infection - HMPV infection is seen in individuals of all ages outside of children ^7^. Nearly all individuals worldwide are seropositive for one or more strains of HMPV, according to sero-epidemiology investigations. HMPV infection causes higher disease severity and high rates of morbidity and mortality in the elderly, although it is usually moderate in otherwise healthy younger persons.^10,11^ Kindly refer to Figure 1 for a brief overview of the disease burden and the epidemiology of HMPV.

## **3.** Virology and Pathogenesis

HMPV and RSV have several characteristics, including non-segmented negative-sense RNA viruses (nsNSVs) from the Pneumoviridae family^12^. HMPV has a 13.3 kb negative-sense RNA genome with eight genes: 3'-N-P-M-F-M2-SH-G-L-5'^13^. Surface glycoproteins are composed of the F, SH, and G proteins. The M and M2 genes use overlapping ORFs to encode the matrix protein M, as well as the M2-1 and M2-2 proteins. The L protein contains RNA-dependent RNA polymerase activity (RdRP), which is responsible for both the transcription of capped and polyadenylated viral mRNAs and genome replication. The viral genome is encapsulated in a sheath of oligomerized copies of the nucleoprotein N, which forms a ribonucleoprotein complex known as nucleocapsid ^13-15^.

The nucleocapsid serves as a template for L's transcription and replication, which also requires the obligatory polymerase cofactor, phosphoprotein P, to create the active L/P holoenzyme ^16^. nsNSVs are distinguished by the creation of specialized viro-induced cytoplasmic inclusions that concentrate viral RNA and proteins. During RSV and HMPV infections, these inclusion bodies (IBs) have been found to contain L/P and N, as well as be active sites of viral transcription and replication ^17,18^.

The N-RNA/P interaction is hypothesized to be critical in several aspects of the viral replication cycle: 1) attaching the L/P complex to the nucleocapsid during transcription/replication, 2) production of IBs, and 3) loading of nucleocapsids into offspring virions via contact with the viral matrix ^19^. Pneumoviruses cause infection by connecting to target cells via surface glycoproteins called the fusion protein (F) and/or the attachment protein (G), which interact with host receptors and attachment factors. Following membrane fusion, F undergoes a significant conformational shift, and the viral nucleocapsids are released into the cytoplasm of the infected cell ^20,21^.

HMPV has been demonstrated to be cell-associated, causing the production of long, actin-based filamentous extensions that are essential for direct cell-to-cell dissemination in vitro, even in the presence of neutralizing antibodies ^22^.

Interferon (IFN) production is an important mechanism for a host's immune system to respond to invading pathogens such as viruses, bacteria, and parasites ^23^. Viral infection induces interferons, which are the first line of innate antiviral immunological defense. HMPV, a respiratory pathogen, predominantly targets epithelial cells of the upper and lower respiratory tracts, as well as lung-resident leukocytes. HMPV infection causes numerous histological abnormalities in the lungs of infected humans and animals. These changes include disruption to the respiratory epithelial architecture, sloughing of epithelial cells, hyaline membrane development, loss of ciliation, and enhanced mucus production and inflammation of the lung interstitium (also known as parenchymal pneumonia or pneumonitis). Indeed, multiple focal lesions have been reported in which inflammation of the lung parenchyma is associated with the presence of HMPV proteins, implying that (i) host immunity is activated only in regions of virus replication and (ii) the degree of respiratory involvement is strongly correlated with viral spread in the respiratory tract. Except for immunocompromised hosts and those with underlying illnesses, who may develop chronic airway inflammation, HMPV respiratory infections are transient and self-limiting. These acute and chronic pathophysiological alterations limit gas exchange and cause respiratory discomfort in infected individuals ^24-26^.

## 4. Clinical Manifestations and Complications

Human metapneumovirus (HMPV) is a serious respiratory disease that affects individuals of all ages, with a special emphasis on children, the elderly, and immunocompromised patients. It frequently causes upper and lower respiratory tract infections, with symptoms ranging from a moderate cold to severe respiratory distress. In pediatric populations, HMPV is the major cause of hospitalization for bronchiolitis and pneumonia, which are frequently accompanied by fever, cyanosis, subcostal retractions, and wheezing. Children's radiographic findings may include hyperinflation and perihilar infiltrates, with severe instances progressing to acute respiratory distress syndrome (ARDS) ^21,27^. HMPV infection in adults typically causes influenza-like symptoms such as fever, cough, nasal congestion, and shortness of breath. While healthy individuals normally get a minor illness, those with underlying disorders such as asthma, chronic obstructive pulmonary disease (COPD), or cardiovascular disease are more likely to develop problems. In older and immunocompromised patients, HMPV can cause severe pneumonia, extended sickness, and a greater risk of respiratory failure, necessitating supplemental oxygen therapy ^28^. HMPV is also a known cause of community-acquired pneumonia (CAP), and it often coexists with other respiratory viruses such as respiratory syncytial virus (RSV) and influenza, complicating clinical diagnosis and management. Common radiographic findings in hospitalized patients include bilateral infiltrates and, in severe cases, ground-glass opacities, which may suggest extensive lung involvement. The virus has a specific seasonal cycle, peaking from late winter to early spring in temperate areas. The clinical presentation of HMPV can be similar to other viral pneumonia, making differential diagnosis difficult. However, its significant link to wheezing and worsening of existing respiratory symptoms can assist in distinguishing it in some cases. Molecular techniques such as reverse transcription polymerase chain reaction (RT-PCR) are used largely for diagnosis, with immunofluorescent antibody tests and serology serving as backup options. Despite its clinical significance, HMPV is frequently underdiagnosed due to symptom overlap with other respiratory viruses ^2,24,29^.

## 5. Diagnostic approaches

Early detection of HMPV infection will aid in the development of efficient disease control strategies, such as containing the epidemic and giving patients prompt medical attention. Therefore, over the years a wide range of technologies and testing techniques have been developed for the detection of HMPV. These include the recently developed CRISPER-CaS12a and meta-genomic next-generation sequencing (mNGS), as well as more traditional techniques like RT-PCR, multiplex-PCR, viral cultures, fluorescent immunoassays, serological assays, and enzyme assays as mentioned in Table 1.

### 5.1. RT-PCR

A type of nucleic acid amplification test (NAAT), reverse transcriptase polymerase chain reaction (RT-PCR) has been the gold standard for the diagnosis of HMPV in the modern era. This technique involves the reverse transcription of RNA into complementary DNA (cDNA) and the amplification of specific DNA targets such as HMPV genes P, M, F, and N using PCR ^30,31^.

### 5.2. Multiplex-PCR

Multiplex PCR (mRT-PCR) creates amplicons that are unique to various target sequences by combining many primer sets into a single PCR tube. For the primer pairs to work at the same annealing temperature, their designs should be optimum. Target-specific probes tagged with various fluorescent dyes are used to identify different amplicons ^30,32^. A wider variety of respiratory viruses can be detected in a single test run in this approach. Additionally, mRT-PCR can be used to gather information about coinfection. Compared to traditional techniques like viral culture or direct fluorescent antibodies, mRT-PCR technologies have been shown to boost the detection of respiratory viruses by 30% to 50%. Several studies have also reported that this method has a sensitivity and specificity of 100% and 96%, respectively, compared to 54.6% and 100% for rRT-PCR ^33-35^.

### 5.3. Viral Culture 

The gold standard for identifying an infection is the isolation of viruses from culture. Studies have reported that the sensitivity and specificity of cell culture detection methods when compared to RT-PCR were found to be 68% and 99% ^35,36^. To isolate viruses, a known cell line is inoculated with an infectious sample from a nasopharyngeal swab or aspirate and then incubated for 7 to 10 days to observe the development of cytopathic effects (CPE). Cell lines commonly employed for isolating viruses like the influenza virus and respiratory syncytial virus include Madin-Darby canine kidney cells, A549 cells, mink lung epithelial cell lines, human lung diploid fibroblast cells (MRC-5 and WI-38), HeLa cells, rhesus monkey kidney cells (LLC-MK2), and buffalo green monkey kidney cells. For HMPV, multiple cell lines such as Vero, HEp-2, Hep G2, 293, and LLC-MK2 have been used over the past decade. However, the most approved ones are the human Chang conjunctiva cell line (clone 1-5C4) and the feline kidney CRFK cell line. Additionally, recent developments have given rise to the innovation of shell vial culture, which comprises inoculating samples onto a cell monolayer in shell vials, centrifuging the samples, and then continuing to incubate them. Antibodies specific to the virus are used to quantify the HMPV antigens after 24 to 48 hours. Rapid identification of slow-growing viruses, such as HMPV, is made possible via shell vial culture ^4,30,31,37^.

### 5.4. Metagenomic next-generation sequencing (mNGS)

Metagenomic next-generation sequencing (mNGS) is a cutting-edge, high-throughput diagnostic technique extensively used for virus genome sequencing,

**Table 1:** Table 1. Diagnostic approaches aimed at early detection of HMPV

Diagnostic Method	Description	Advantages	Limitations
RT-PCR (Reverse Transcriptase Polymerase Chain Reaction)	Gold standard NAAT test that amplifies HMPV genes (P, M, F, N)	High sensitivity and specificity	Requires specialized equipment
Multiplex-PCR	Uses multiple primers in a single test to detect various respiratory viruses	Detects co-infections; 30-50% higher detection than traditional methods	Requires optimized primer design
Viral Culture	Traditional method where the virus is grown in cell cultures	High specificity (99%)	Slow (7–10 days); lower sensitivity (68%)
Shell Vial Culture	A rapid version of viral culture using centrifugation and antigen detection	Faster than traditional culture (24-48 hrs)	Requires virus-specific antibodies
mNGS (Metagenomic Next-Generation Sequencing)	High-throughput sequencing for viral genome analysis	Detects novel pathogens; high sensitivity (80%)	Expensive; requires advanced lab facilities
Fluorescent Immunoassay (DFA)	Uses fluorescently labeled antibodies for direct antigen detection	Fast results; can be combined with viral culture	Lower sensitivity compared to PCR
Viral Neutralization Assay	Detects virus-specific antibodies produced in response to infection	High sensitivity and faster than traditional methods	Requires live virus and biosafety facilities
Enzyme Immunoassay (EIA/ELISA)	Uses enzyme-conjugated antibodies to detect viral antigens	Colorimetric detection; commonly used in clinical labs	May have cross-reactivity; requires careful validation

discovering novel pathogens, and various research applications. It also enables the amplification of entire viral genomes with high precision. However, RNA genomes must first be reverse - transcribed into cDNA before employing subsequent methods. Studies have demonstrated that mNGS has an 80% sensitivity rate for detecting HMPV, which is a promising outcome, though further advancements are required to enhance its accuracy ^31,38^.

### 5.5. Fluorescent Immunoassay 

DFA testing, one of the many fluorescent immunoassays, uses fluorescently labeled antibodies to directly detect the presence of particular antigens and has been used extensively in clinical laboratories to identify viruses. This method uses fluorescently tagged, virus-specific antibodies, and respiratory epithelial cells obtained from nasopharyngeal swabs or aspirates are directly stained with this technique. DFA techniques are also commonly used alongside shell vial culture or viral culture to increase sensitivity and specificity. The sensitivity of viral detection has also been improved by using a modified cytospin-enhanced DFA ^30^. Studies have demonstrated that cytospinning is 85.4% more effective than other techniques because it reduces insufficient smears and enhances cell morphology, which leads to improved performance ^6^.

### 5.6. Viral-Neutralization Assays 

The recognition of virus-specific antibodies produced by viral infection is accomplished by virus-neutralization tests. The immune system produces antibodies that are particular to viruses to neutralize them. Moreover, this approach takes less than a week to complete and has demonstrated greater sensitivity and quicker turnaround times than traditional procedures ^30^.

### 5.7. Enzyme Immunoassays

The primary viral antigen-antibody combination is bound by secondary/ detection antibodies that have been conjugated to enzymes. The enzyme catalyzes the creation of a colorful end product that can be seen and measured after the proper substrate is added and allowed to incubate. β-galactosidase, horseradish peroxidase, and alkaline phosphatase are the most commonly utilized enzymes ^4,30,31^ .

## 6. Current management options

Human Metapneumovirus (HMPV) is currently treated mostly with supportive care because no particular antiviral drug has been approved. Management techniques are aimed at relieving symptoms and minimizing consequences, especially in high-risk groups such as the pediatric population, immunocompromised individuals, and the elderly. Supportive treatment remains the cornerstone of HMPV management. Patients suffering from respiratory distress, particularly those with bronchiolitis or pneumonia, are frequently given oxygen therapy. In extreme situations of respiratory compromise, mechanical ventilation or non-invasive respiratory assistance, such as a high-flow nasal cannula (HFNC) or continuous positive airway pressure (CPAP), may be required. Hydration support is also important, especially in pediatric patients who may have reduced oral intake due to respiratory distress ^3,39,40^.

### 6.1. Ribavirin and other antiviral agents

Ribavirin, a broad-spectrum antiviral, has shown in vitro action against HMPV and has been utilized in a few circumstances, notably in immunocompromised patients. However, clinical efficacy data are sparse, and routine use is not advised due to probable toxicity. In a mouse study, ribavirin injected intraperitoneally at 40 mg/kg twice daily dramatically reduced HMPV replication in the lungs by around 5 log₁₀ and decreased pulmonary inflammation. Mice treated with ribavirin lost less weight than untreated controls, suggesting a potential therapeutic effect. Another rodent study on HMPV infection found that those treated with phosphate-buffered saline (PBS) lost 16.3% ± 6.81% of their weight by day 5, whereas control animals gained 4.3% ± 1.15 percent. Ribavirin-treated mice dropped 3.7% ± 3.27% of their body weight, which stabilized between days 2 and 3 for those receiving ribavirin or corticosterone. Moreover, on day 5, mice treated with ribavirin lost 10.8% ± 7.76%, those treated with corticosterone lost 7.7% ± 5.87%, and the combination group lost 6.1% ± 3.48% of their weight. Ribavirin treatment resulted in significantly reduced viral loads (4.34 × 10² TCID50/g) and combination groups (1.25 × 10² TCID50/g) compared to untreated mice. Histopathology demonstrated that ribavirin-treated animals had less lung inflammation, but corticosterone alone reduced interstitial and alveolar inflammation without changing virus loads. In addition, ribavirin efficiently reduced viral replication and inflammation, whereas corticosterone reduced inflammation and weight loss, implying a possible advantage when paired with antiviral medication. Further research should look into different glucocorticoids and therapy regimes. Other investigational antiviral medicines, including fusion inhibitors that target the viral F protein, have shown promise in preclinical trials but are not yet ready for clinical use ^37,41-46^. Kindly refer to Table 2 for a systemic summary of studies assessing ribavirin as a management regime for HMPV.

### 6.2. Immunomodulatory therapies

Corticosteroids have been evaluated in severe cases of airway inflammation and wheezing, especially in patients with underlying asthma or chronic lung illness. However, the evidence for their routine usage in HMPV infections is equivocal. Intravenous immunoglobulin (IVIG) and convalescent plasma therapy have been investigated in immunocompromised patients, with some results indicating possible benefits in lowering illness severity ^44,47^.

### 6.3. Monoclonal Antibody Therapy

Palivizumab, a monoclonal antibody used for respiratory syncytial virus (RSV) prevention, has been shown to cross-react with HMPV in experimental studies. However, it is not yet licensed for HMPV prevention or treatment. Monoclonal antibodies targeting HMPV are currently being developed, and preclinical results are promising ^48,49^.

### 6.4. Antimicrobial Use and Secondary Infections

Although HMPV is a viral pathogen, it can cause secondary bacterial infections, especially in hospitalized patients. In cases of suspected bacterial superinfection, empirical antibiotic therapy may be attempted; nevertheless, routine antibiotic usage in uncomplicated viral infections is discouraged ^50,51^.

## 7. Vaccines and Preventive Measures

Although many attempts have been made to provide a safe and effective vaccine, there are presently no approved vaccinations for HMPV ^4,35^. Nonetheless, several encouraging live-attenuated vaccines have been developed. Numerous HMPV vaccination candidates have been tested in non-human primate and rodent models. None have been tested on human volunteers yet, despite their encouraging results ^35^.

### 7.1. Live-Attenuated Vaccines

The live particles used to give vaccine recipients protective immunity against particular viruses are known as live attenuated viruses. To replicate a natural infection and enable the host immune system to identify the virus as a whole, these vaccines contain viruses that have been sufficiently reduced to lose their virulence while retaining their structural integrity. As a vaccine, these viruses stimulate humoral and cellular immunity and don't need a booster shot. Both recombinant and non-recombinant vaccinations are possible. These vaccines are both recombinant and non-recombinant ^7,52^.

According to a few animal studies performed on hamsters, a cold-adapted, non-recombinant, live-attenuated HMPV vaccination might offer hamsters total protection. By transfecting the cultured animal cells with viral cDNA and plasmids encoding the virus-specific RdRP, multiple attempts have also been undertaken to produce recombinant HMPV. Genes encoding G, SH, M2-2, or P protein could be deleted to produce attenuated HMPV. Although live-attenuated vaccinations did not completely protect against virus replication, a subsequent study on cynomolgus macaques showed elevated antibody levels following immunization ^7,53,54^.

A live attenuated vaccine strain of HMPV was created in a recent study by altering the F protein's glycosylation location. It was discovered that this vaccination provided full protection against challenges from homologous viruses and partial protection against challenges from heterologous viruses ^55^.

### 7.2. Inactivated Vaccines

Given that inactivated vaccines are stable and biologically safe, they have shown great efficacy in treating a variety of viral infections, including polio and influenza ^7^. When challenged with the wild-type virus, immunization with formalin-inactivated HMPV has been shown to increase illness severity which may be due to increased pulmonary histopathology and an unbalanced immune response with elevated levels of Th2 cytokines such as IL4. Furthermore, heat-inactivated HMPV vaccination in animal models has also been demonstrated to be harmful, as seen by increased Th2 cytokine levels and eosinophil infiltration in the vaccinated animals' lungs ^56,57^. However, on the other hand, it has been shown that in recent years, inactivating viruses like RSV using nanoemulsions can produce safe vaccines that can boost the host body's ability to eliminate the virus and elicit an efficient humoral immune response ^58^.

### 7.3. Subunit Vaccines

It has been demonstrated that vaccination with partial or full-length viral proteins, as opposed to the entire virus, is adequate to reduce viral pathogenesis. This includes viral vectors and virus-like particles (VLPs) ^7,35^.

### 7.4. Viral Vectors

Recombinant proteins have traditionally been introduced and expressed in targeted hosts using viral vectors. Studies performed on rodents have shown that when a retroviral vector encoding the HMPV F protein has been administered intraperitoneally, it has demonstrated to elicit a robust and protective immune response against several HMPV subtypes; however, this was not the case for the HMPV G protein ^59^. Furthermore, African Green Monkeys showed a significant degree of immunogenicity and protective effectiveness against HMPV infection when HMPV F protein was expressed using Venezuelan Equine Encephalitis virus-based Viral Replicon Particle (VEE-VRP) ^48^. Similarly, cotton rats who were infected with a recombinant Sendai virus that expressed the shortened fusion protein of HMPV developed neutralizing antibodies, suggesting that the recombinant could shield the vaccinated host from HMPV infection ^60,61^.

**Table 2:** Table 2. Summary of studies assessing Ribavirin as an antiviral management regime for HMPV.

Year	Study Type	Study Description	Findings	Indication
2005	Mouse Model Study (PMC, 2005) ^44,65^	Experimental study on BALB/c mice, administered intraperitoneal ribavirin.	Ribavirin reduced HMPV replication by 5 log₁₀ and decreased lung inflammation and weight loss compared to untreated controls.	Evaluation of ribavirin’s antiviral effects in HMPV-infected mice.
2007	Case Report (JHLT, 2007) ^66,67^	Case report on the use of intravenous ribavirin in a patient with severe HMPV infection.	Ribavirin resulted in improvement and recovery of the patient, although further studies are needed.	Use of intravenous ribavirin in severe HMPV infections.
2017	Clinical Practice Guidelines (ASTCT, 2017) ^68,69^	Guidelines on the use of ribavirin in viral respiratory infections, including RSV and HMPV, particularly in immunocompromised adults.	Ribavirin has been used for treating RSV and HMPV infections in immunosuppressed adults with some benefits but is not standard practice.	Treatment of HMPV and RSV infections in immunosuppressed patients.
2020	Lung Transplant Recipients Study (PubMed, 2020) ^70^	Retrospective review involving 71 patients (47 with RSV and 24 with HMPV).	Ribavirin reduced the risk of progression from URTI to LRTI in HMPV patients. No significant effect on mortality or mechanical ventilation.	Ribavirin for high-risk lung transplant recipients with HMPV infection.
2020	In Vitro Study (eMedicine, 2020) 71	Laboratory-based study assessing ribavirin’s antiviral activity against HMPV in cell cultures.	Ribavirin demonstrated antiviral activity against HMPV in vitro.	Testing ribavirin’s efficacy against HMPV in vitro.

### 7.5. Virus-like-particles (VLPs)

Virus-like particles (VLPs) have proven to be effective vaccine candidates against several significant viral diseases, including rotavirus and human papillomavirus. VLPs are one of the most effective immunization methods due to their nonpathogenic nature and capacity to reveal viral proteins in their original shape. By producing the HMPV fusion and matrix protein in suspension-adapted HEK293 cells, Cox and his colleagues created an HMPV VLP and were able to elicit a protective immune response in a mouse model without any Th2-biased cytokine response. Even though an HMPV-VLP vaccine appears to be a promising strategy, further study is necessary to create a vaccine that will work against every hMPV subgroup ^7,62^.

### 7.6. Epitope Based Vaccines

To determine the immunogenic epitopes of HMPV, numerous investigations have been conducted ^35,63^. Major histocompatibility class I restricted CTL epitopes on the N, G, M2-2, and SH proteins of HMPV were identified by multiple researchers. Experimental studies also showed that administering these epitopes as a peptide vaccine results in effector and memory CTL responses, as well as increased expression of Th1 type cytokines (IFNc and IL12) and a significantly lower level of Th2 type cytokine response (IL10 and IL4), in a mouse model of lung disease. Immunomodulation by HMPV challenge has been demonstrated to be decreased by T cell epitope vaccinations. Moreover, following an HMPV challenge, mice inoculated with an HMPV cytotoxic T cell epitope vaccine generated fewer Th1 and Th2 type cytokines than mice who were not immunized ^63-71^.

## 8. Future Directions in outbreak prevention and management

### 8.1. Monoclonal antibody and Polyclonal antibodies

Immunomagnetic positive selection (Miltenyi) was used to isolate CD22+ B cells from donor peripheral blood mononuclear cells. CpG and recombinant human interleukin 2 (IL-2) were used to activate the cells for five days. The cells were then seeded onto irradiated, IL-2-supplemented feeder cells at a density of 20 cells/well after being negatively immune magnetically chosen using antibodies against immunoglobulin M (IgM), immunoglobulin D, and immunoglobulin A. Epstein-Barr virus transformation was performed. After two weeks, the enzyme-linked immunosorbent assay (ELISA) was used to analyze the supernatants against the recombinant HMPV B2FΔTM protein. From oligoclonal B-cell cultures that corresponded to antigen-reactive wells, variable-heavy, and variable-light chain genes were cloned, and ELISA was used to verify the specificity of the cloned antibodies ^72^. Human mAb against HMPV F is called DS7. As previously reported, expression plasmids expressing 54G10 or DS7 were transfected ^73^.

### 8.2. Development of targeted therapies

Serial twofold dilutions of each peptide were mixed with TCID50 of HMPV strain C-in infection medium and then added to LLC-MK2 cells. After a few hours, the medium was removed and fresh medium containing serial twofold dilutions of peptides was added after a few days, supernatants were collected and stored at −80°C until quantification by a real-time reverse transcriptase-PCR assay for the N gene using previously described primers and a new TaqMan probe (5′-FAM-CTTGRTGCAATGATGAYGGTGTCACTGCXT-Tamra-PH-3′).

Immunostaining was also used to assess the inhibitory activity of the HRA2 peptide against all four HMPV subgroups. Briefly, confluent LLC-MK2 cells were infected with PFU of HMPV and serial dilutions of the peptide the medium was then removed, and an overlay containing methylcellulose and serial twofold dilutions of the peptide was added after which the cell monolayers were immunostained after being fixed with formalin. Every 50% inhibitory concentration (IC50) value was computed from triplicate experiments. The HMPV subgroup A1 fusion events that the HRA2 peptide blocked were subsequently identified by immunostaining. The peptide was supplied during viral adsorption and/or in the methylcellulose overlay, however, the experiment was carried out as previously mentioned ^42^.

### 8.3. The potential of Pan - Pneumovirus vaccines for cross-protection with RSV

RSV and HMPV F proteins' essential immunological characteristics are maintained by RHMS-1. Monoclonal antibodies that target certain antigenic sites bind to RHMS-1 with kinetics comparable to those of their respective targets, as shown by the ELISA and BLI experiments ^74^. mAbs like D25, hRSV90, and motavizumab demonstrated similar binding to RHMS-1 for RSV F. Likewise, mAbs such as DS7, MPV196, and MPV481 bind RHMS-1 with comparable affinity for HMPV F ^75-79^. Epitopes spanning RSV F, HMPV F, and RHMS-1 were detected by cross-reactive mAbs such as MPE8 and 101F, RHMS-1 was not bound by antibodies such as MPV364 and MPV458, which target distinct HMPV-specific locations, in accordance with structural categorization. The conformational architectures of important antigenic regions from both F proteins are typically preserved by RHMS-1. B cells that have previously been exposed to RSV F or HMPV F identify RHMS-1, indicating conserved epitopes. Serum IgG binding to RSV F and RHMS-1, as well as HMPV F and RHMS-1, were positively correlated, according to ELISA analysis of human subjects' plasma IgG responses ^79,80^.

Stimulated peripheral blood mononuclear cells were used to investigate B cell responses at the cellular level. To encourage activation and IgG release, these cells were co-incubated with NIH 3T3 cells that expressed human CD40L, IL-21, and BAFF. The majority of RSV F-positive B cells were also able to identify RHMS-1, according to the results, since RHMS-1 preserves the head of RSV F, a significant B-cell target. Similarly, whereas HMPV F-reactive B cells were typically less common, HMPV F-positive B cells also reacted to RHMS-1. This led to some RHMS-1-positive but HMPV F-negative B cell populations. These findings suggest that RHMS-1 retains critical epitopes from both RSV F and HMPV F proteins, making it recognizable to the human immune system. Strong neutralizing antibodies and cross-protection against RSV and HMPV were produced in mice that received RHMS-1 immunization. BALB/c mice were challenged with either RSV or HMPV after being vaccinated with RHMS-1, RSV F DsCav1, HMPV B2 F monomer, or PBS. Serum IgG binding to both RSV and HMPV F proteins was created by RHMS-1-immunized animals, which also killed representative viruses from each HMPV genotype and RSV subgroup. On the other hand, HMPV B2 F produced non-neutralizing IgG against RSV, whereas RSV F DsCav1 did not produce any cross-reactive IgG. In contrast to single-virus vaccinations, which only protected one virus, RHMS-1 completely protected mice against both RSV and HMPV following a viral challenge ^72,80-82^.

## 9. Conclusion

Human metapneumovirus (HMPV) is a prominent yet underdiagnosed respiratory pathogen that causes severe morbidity in the juvenile, geriatric, and immunocompromised populations. Despite advances in diagnostic methods such as RT-PCR and next-generation sequencing, restricted treatment options impede successful management. Current treatment options are mostly supportive, focusing on oxygen therapy and hydration, although investigational antiviral treatments like ribavirin and monoclonal antibodies show promise. To reduce HMPV's global impact, effective vaccines, including live-attenuated, inactivated, and subunit-based formulations, must be developed urgently. Recent research has focused on pan-pneumovirus vaccines that can provide cross-protective immunity against both HMPV and RSV, potentially revolutionizing preventive techniques. Future research should concentrate on developing targeted antiviral medicines, optimizing vaccine candidates for human trials, and improving global surveillance systems to forecast outbreak trends. Given the seasonal changes and growing acknowledgment of HMPV as a serious respiratory pathogen, a multidisciplinary strategy that combines epidemiology, molecular virology, and immunology is required. Improving public health awareness and early diagnostic measures will be crucial to reducing illness effects.
